# Knee hyperextension is a sign of through‐range laxity and may be effectively managed without routine joint line distalisation in a robotic functional alignment workflow

**DOI:** 10.1002/ksa.70203

**Published:** 2025-12-01

**Authors:** Anton Lambers, Serene Lee, Kate Langton, Dermot Collopy, Gavin Clark

**Affiliations:** ^1^ Medical School The University of Western Australia Perth Western Australia Australia; ^2^ Department of Orthopaedics Northeast Health Wangaratta Wangaratta Victoria Australia; ^3^ Perth Hip and Knee Clinic Perth Australia; ^4^ St John of God Subiaco Murdoch and Midland Hospitals Perth Western Australia Australia

**Keywords:** functional alignment, gap assessment, hyperextension deformity, robotic‐assisted total knee arthroplasty, total knee arthroplasty

## Abstract

**Purpose:**

Traditional management of hyperextension deformity in total knee arthroplasty (TKA) involves routine under‐resection of distal femoral bone to lower the joint line and close the extension gap. This study aimed to assess how a robotic functional alignment (FA) workflow with pre‐resection gap assessment managed hyperextending knees to achieve balance compared to controls.

**Methods:**

A retrospective analysis of a prospective registry was performed using a single implant/robotic platform, two surgeons, between 2016 and 2023. 100 hyperextending TKA patients with hyperextension of 5° or more were compared to a control group of 1881 patients with 0°–10° of fixed flexion contracture.

**Results:**

The hyperextension group had a greater maximum flexion ROM (137° vs. 132°) compared to controls. All four mean start‐of‐case gaps (medial and lateral in flexion and extension) were larger for the hyperextension patients by 0.7–1.2 mm. The femoral joint line was not routinely distalised by a clinically significant amount in the hyperextension patients (0.3 mm difference in means; 0.6 mm vs. 0.3 mm. The hyperextension cohort had relative tibial under‐resection (4.8 mm vs. 5.4 mm; *p* < 0.001) and additional insert thickness (11.2 vs. 10.3 mm; *p* < 0.001) resulting in an overall mean tibial joint line elevation of 1.5 mm greater than controls. At the 1‐year mark, there was no difference between groups when assessing range of motion (*p* > 0.05) and forgotten joint score (FJS, *p* > 0.05). Two patients had hyperextension recurrence at 1‐year review, measured at –2° and –5°.

**Conclusions:**

Hyperextending arthritic knees may not require routine distalisation of the femoral joint line to achieve balance and close the extension gap. These knees have a global laxity that can often be effectively managed with predominantly tibial sided changes.

**Level of Evidence:**

Level III, therapeutic retrospective comparative study.

## Graphical abstract

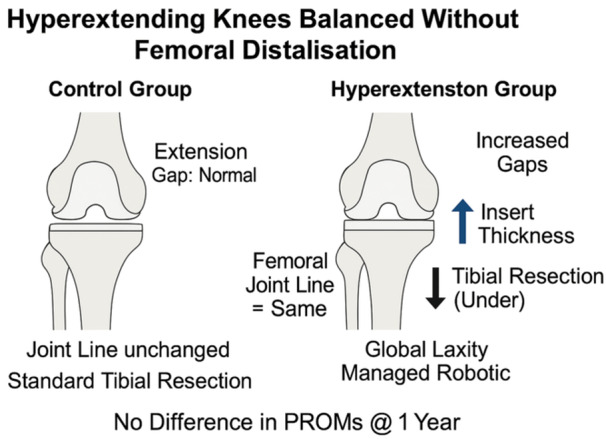

**Abbreviations**
aHKA: arithmetic hip knee ankle angle
BMI: body mass index
CON: controls
CR: cruciate retaining
CS: condylar stabilised
CT: computed tomography
FA: functional alignment
FJS: forgotten joint score
HYP: hyperextension patients
PROMs: patient reported outcome measures
PS: posterior stabilised
ROM: range of motion
SD: standard deviation
TKA: total knee arthroplasty

## INTRODUCTION

Knee hyperextension challenges the planning and execution of total knee arthroplasty (TKA) [6]. Definitions of the term knee hyperextension vary between 1° and 10° of recurvatum and is most often referred to as being 5° or more [6, 11, 13, 14]. While long term functional outcomes have been demonstrated to be worse in patients with residual hyperextension after TKA, most patients with pre‐operative hyperextension have little to no residual hyperextension after surgery [10, 11, 14, 16, 17, 18]. Risk varies with implant design—particularly single‐radius constructs—and cohort characteristics reported in specific series [9, 12, 20]. To avoid post‐operative hyperextension the traditional teaching for instrumented, mechanically‐aligned resections would encourage an under‐resection of bone, particularly the distal femur, to close the extension gap [8]. This is based on the biomechanical assumption that hyperextension is a laxity issue that is isolated to full extension. It is possible that laxity in flexion is not noted as a corresponding feature of the hyperextending knee phenotype due to difficulty in clinical assessment or quantification clinically prior to surgery [15].

Robotic total knee replacement using a functional alignment (FA) philosophy focuses on quantitative gap measurements and computed tomography (CT) based resection measurements and has the potential to change the way hyperextension is managed using pre‐resection balancing [4]. We sought to assess the way a robotic‐assisted FA technique manages hyperextending knees compared to a group of non‐hyperextending controls. Seo et al. reported similarly with 1.7 mm more insert thickness and only 0.8 mm less distal femoral resection in hyperextension patients in their cohort [19]. We hypothesised that hyperextension patients would be balanced mostly by tibial joint line changes due to large both extension and flexion gaps. The primary outcome was 1‐year Forgotten Joint Score (FJS) and secondary outcomes included 1‐year range of motion (ROM) and intra‐operative gaps and resection depths.

## MATERIALS AND METHODS

A retrospective analysis of patients in a prospective clinical knee arthroplasty registry was conducted. Patients underwent primary TKA (Stryker Triathlon, Mahwah, New Jersey, USA) enabled with the Mako robotic system (Stryker, Fort Lauderdale, Florida, USA) by two senior arthroplasty surgeons between 20 December 2016 and 31 December 2023. Patient cohorts were defined as controls (CON) if the start‐of‐case on table recorded extension was between 0° and 10° and hyperextension patients (HYP) if the start‐of‐case extension was 5° or greater of hyperextension. Start‐of case extension was measured under anaesthesia using the robotic system after anatomical registration to the CT model. The patient′s limb was lifted by the foot and the maximum resting extension was measured. Five degrees of hyperextension was chosen as the cut off as a clinical figure based on previous literature published on the subject [13, 14]. A total of 2906 total knee replacements performed in 2457 patients were evaluated for start‐of‐case extension. 1981 TKAs in 1730 patients were included in the study based on the cohort extension parameters, with 100 hyperextension and 1881 control TKAs.

All patients received a cruciate retaining (CR) or posterior stabilised (PS) femur with a single radius of curvature and a cruciate retaining (CR), condylar stabilised (CS) or posterior stabilised (PS) insert (TKA1.0 and 2.0 software and Triathlon Total Knee implant; Stryker, Mahwah, New Jersey, USA). The FA technique and alignment limits used in this study have previously been described [3]. Data was collected prospectively and included preoperative patient demographic details, ROM and Forgotten Joint Score (FJS). Data such as start‐ and end‐of‐case gaps, robotic ROM measurements, bone resections and insert thickness were recorded intra‐operatively. Patients were reviewed at 1 year post‐surgery for assessment of ROM and FJS [1].

The changes to the height of the femoral and tibial articular surfaces were defined as follows. Femoral joint line distalisation and tibial joint line elevation were used to describe additional implant thickness beyond the thickness of the resected cartilage plus bone. Each measurement represented the average of the medial and lateral resections. The cartilage thickness was presumed at 2 mm and this was added to the CT‐based resections which were measured referencing bony landmarks [7]. A limitation of this is it cannot account for bone loss. For femoral changes this was the distal femoral implant thickness (8.5 mm) minus the sum of the bone resection plus 2 mm. Tibial joint line elevation had two contributors. Tibial under‐resection involves removing less bone and cartilage than is replaced by the thinnest tibial implant combination (tray and insert) of 9 mm in this system. The contribution from the insert was calculated as the insert thickness beyond the thinnest available.

A two‐sided independent samples t‐test was used to compare means of parametric data and a two‐sided Mann–Whitney *U* test was used for non‐parametric data with continuity correction and an alpha of 0.05. For power calculation, the primary outcome of FJS at 1‐year was chosen, which has a minimal clinically important difference (MCID) of 17 [5]. The mean and standard deviation of 1 year FJS in our clinical registry is 71.3 ± 26.3. This provided a power calculation of 404 patients required (21 hyperextension patients, 383 controls).

Quantitative data were analysed using Microsoft Excel (Excel for Mac 2016; Microsoft). Quantitative data were analysed using Microsoft Excel (Excel for Mac 2016; Microsoft). Statistical analysis was conducted using SPSS (IBM SPSS Statistics, Version 29.0, Released 2023: IBM). The study was conducted in accordance with the World Medical Association Declaration of Helsinki and ethics approval was granted by the hospital group HREC (SJGHC HREC ref1388).

## RESULTS

### Demographics and preoperative data

**Figure 1:**
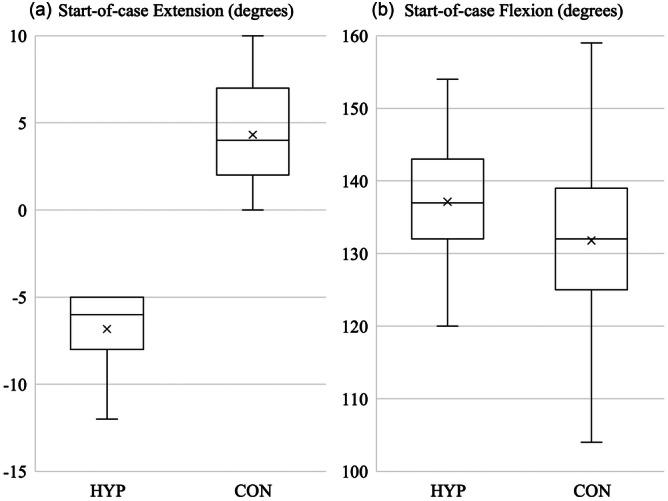
Start‐of‐case knee range measured intra‐operatively by the robotic encoder. (a) Extension. (b) Flexion (degrees). CON, controls; HYP, hyperextension patients.

**Figure 2:**
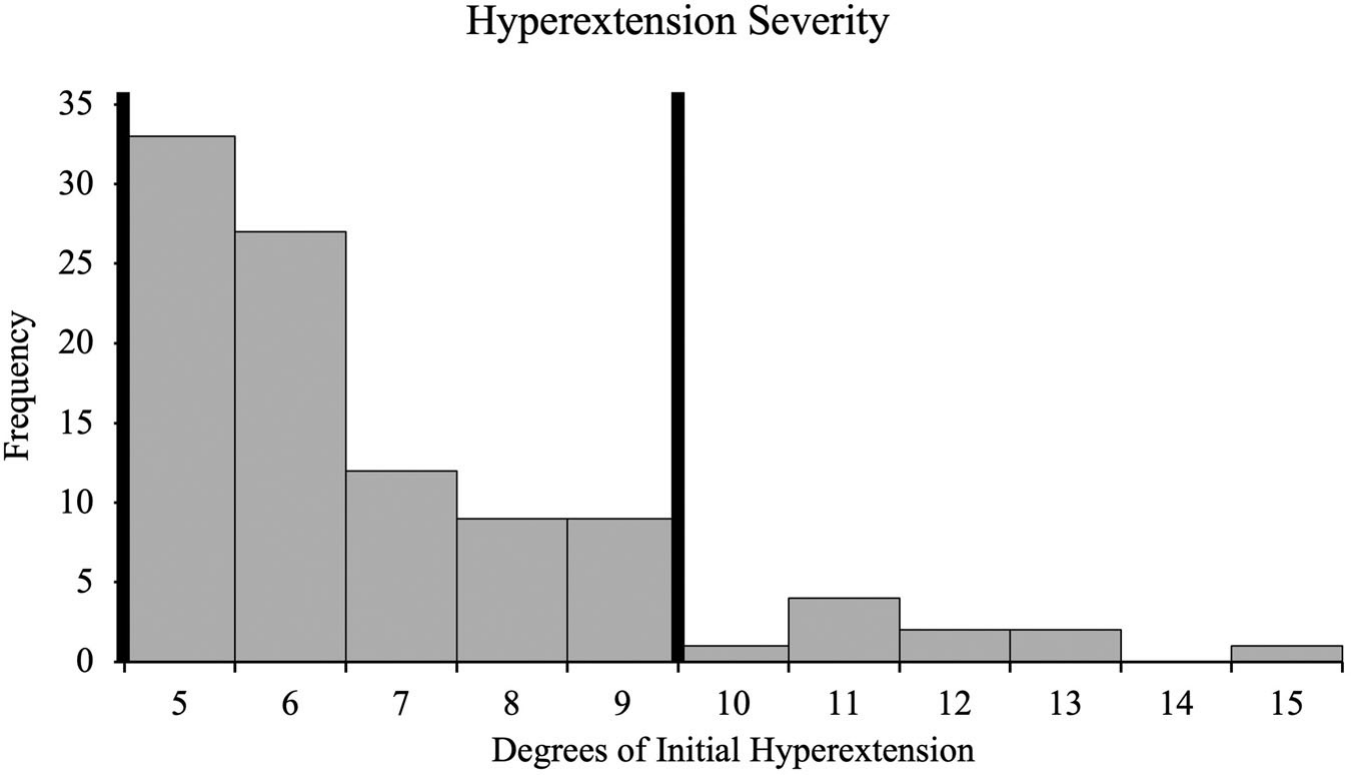
Distribution of start‐of‐case extension in the HYP cohort (*n* = 100). Bin width = 1°. Vertical lines mark 5° (inclusion threshold) and 10° (severe hyperextension). HYP, hyperextension cohort.

The hyperextension group had a greater start‐of‐case flexion ROM (137 vs. 132 degrees, *p* < 0.001) as demonstrated in Figure 1. The hyperextension group also demonstrated a lower BMI and had a less varus arithmetic hip knee ankle angle (pre‐ and post‐operatively) when compared to controls. Demographic data is summarised in Table 1. The range of hyperextension values present is shown in the Figure 2 histogram.

**Table 1:** Demographics and preoperative data.

	HYP (*N* = 100)	CON (*N* = 1881)
Age (years)	68.3 ± 8.8	69.1 ± 8.9
Gender (%F)	57%	50%
BMI (kg m^−2^)	29.1 ± 5.3	30.8 ± 5.7
Initial aHKA	–0.4° ± 2.4°	–1.2° ± 2.5°
Final aHKA	–1.4° ± 2.2°	–2.1° ± 2.3°
Initial extension	–6.8° ± 2.2°	4.3° ± 3.0°
Initial flexion	136.8° ± 8.1°	132.4° ± 9.7°
Liner type:		
CR	29 (29%)	654 (36%)
CS	70 (70%)	1143 (62%)
PS	1 (1%)	31 (2%)

*Note*: Data presented as mean ± SD.

Abbreviations: aHKA, arithmetic hip knee ankle angle; BMI, body mass index; CON, control group; CR, cruciate retaining; CS, condylar stabilising; HYP, hyperextension cohort; PS, posterior stabilising; SD, standard deviation.

### Gaps

**Figure 3:**
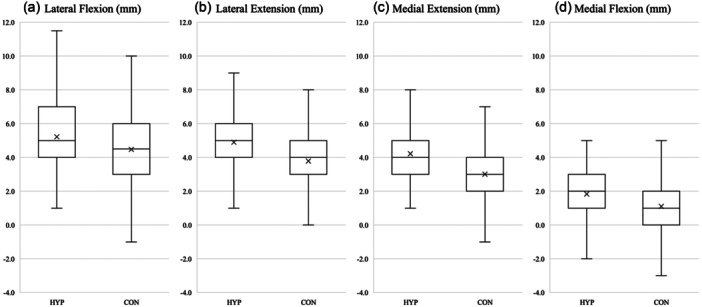
Start‐of‐case gaps by group. (a) Lateral flexion (mm). (b) Lateral extension (mm). (c) Medial extension (mm). (d) Medial flexion (mm).

**Figure 4:**
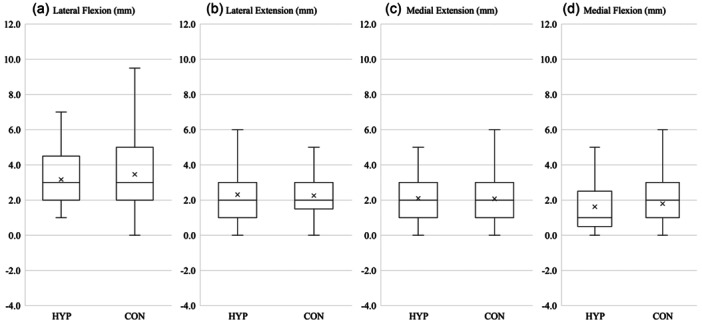
End‐of‐case gaps by group. (a) Lateral flexion (mm). (b) Lateral extension (mm). (c) Medial extension (mm). (d) Medial flexion (mm). CON, control group; HYP, hyperextension group.

The mean start‐of‐case gaps were larger for the hyperextension patients compared to controls in all four gap measurements by 0.7–1.2 mm (*p* < 0.001, Figure 3, Table 2). After implantation, the mean end‐of‐case gaps corrected to a similar mean (Figure 4, Table 2). The four gap measurements were also compared between a cohort of severe hyperextension patients (10° or greater, 10 patients) and controls. The same pattern of overall increased laxity was observed and the details of these severe patients are summarised in Supporting Information: Appendix A.

**Table 2:** Initial and final mean gaps by group (mm, mean and SD) initial.

	Initial			Final		
	HYP	CON	Diff. (95% CI)	HYP	CON	Diff. (95% CI)
Lateral flexion	5.2 ± 1.9	4.5 ± 1.9	0.7 (0.3–1.1)	3.2 ± 1.7	3.5 ± 1.7	–0.3 (–0.6 to 0.0)
Lateral extension	4.9 ± 1.6	3.8 ± 1.5	1.1 (0.8–1.4)	2.2 ± 1.2	2.2 ± 1.0	0.0 (–0.2 to 0.2)
Medial extension	4.2 ± 1.4	3.0 ± 1.6	1.2 (0.8–1.5)	2.1 ± 1.3	2.1 ± 1.0	0.0 (–0.2 to 0.2)
Medial flexion	1.8 ± 1.5	1.1 ± 1.8	0.7 (0.3–1.0)	1.6 ± 1.3	1.8 ± 1.2	–0.2 (–0.4 to 0)

Abbreviations: CI, confidence interval; CON, control group; Diff, difference; HYP, hyperextension group; SD, standard deviation.

### Resections and joint line changes

No clinically relevant deliberate bony under‐resection of the distal femur was seen in hyperextension patients with a difference of 0.3 mm between the HYP and CON group means (5.9 mm vs. 6.2 mm, *p* < 0.001). There was no difference in lateral posterior condyle resection (mean 6.6 mm vs. 6.5 mm, *p* = 0.425) or medial posterior condyle resection (mean 8.8 mm vs. 8.6 mm, *p* = 0.130). Hyperextension patients had an average of 0.6 mm less tibial resection (mean 4.8 mm vs. 5.4 mm; *p* < 0.001) and additional 0.9 mm of insert thickness (mean 11.2 vs. 10.3; *p* < 0.001) giving an overall mean tibial joint line elevation of 1.5 mm greater than controls.

**Figure 5:**
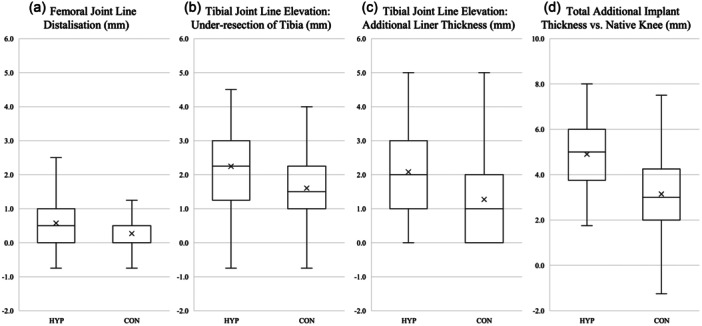
Joint line changes by group. (a) Femoral joint line distalisation (mm). (b) Tibial joint line elevation from under‐resection (mm). (c) Tibial joint line elevation from additional insert (mm). (d) Total additional implant thickness vs. native knee (mm).

In both cohorts there was on average both femoral joint line distalisation and tibial joint line elevation to close the gaps (Figure 5). The mean changes were greater in the hyperextension cohort compared to controls across all locations (femoral joint line distalisation, 0.6 vs. 0.3 mm; tibial under‐resection, 2.2 vs. 1.6 mm; additional insert thickness, 2.2 vs. 1.3 mm; total change, 5.0 vs 3.2 mm, all *p* < 0.001).

**Figure 6:**
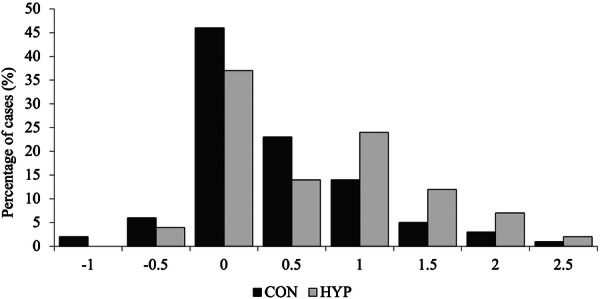
Distribution of femoral joint‐line distalisation (mm) by group. CON, control group; HYP, hyperextension group.

Joint line distalisation of 2 mm or greater occurred in 7 (7%) of the hyperextension cohort and 39 (2%) of the control group (Figure 6). Of these seven patients, five were distalised by 2 mm, one by 2.3 mm and one by 2.5 mm. The relative contribution of the tibial and femoral cuts and the insert thickness was similar between groups with the build‐up occurring predominantly on the tibial side (Figure 5).

### Functional outcomes and ROM

**Figure 7:**
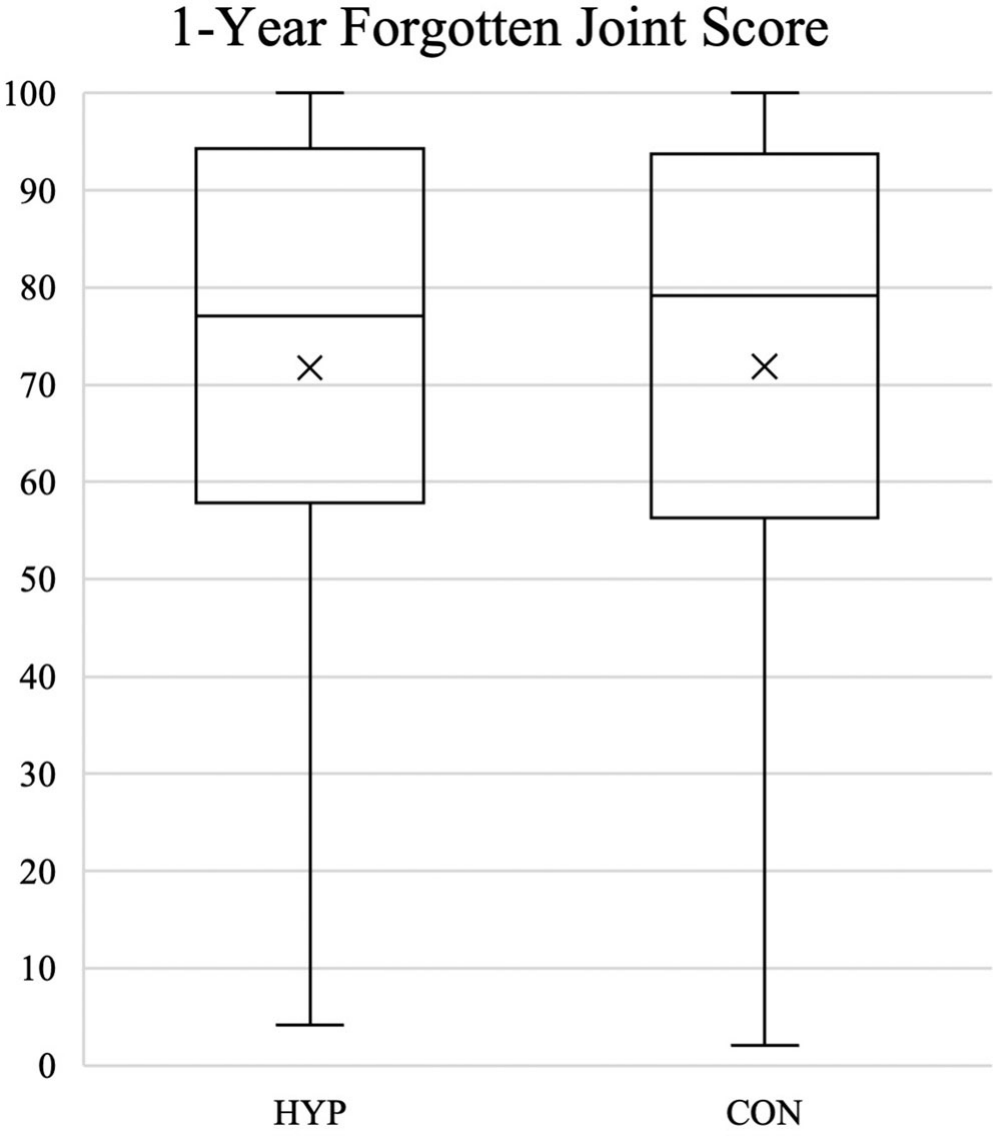
Box‐and‐whisker plot of Forgotten Joint Score results between groups at 1‐year post‐operatively. CON, control group; HYP, hyperextension group.

At the 1‐year mark the Forgotten Joint Score (FJS‐12) results were available for 84% of study cases and demonstrated no difference between cohorts (*p* = 0.996, Figure 7) according to the MCID of 17 [5]. The mean FJS was 71.7 in the hyperextension group (SD 26.0; 95% CI 20.8–112.5) and 71.9 in the control group (SD 25.6; 95% CI 21.6–114.3).

Follow‐up rates were similar between cohorts and baseline demographics varied little between those lost to follow up when compared to those retained (Table 3).

**Table 3:** Baseline demographic data by loss to follow‐up status (1‐yr FJS).

	Retained	Lost
Hyperextenders (%)	78%	22%
Controls (%)	89%	11%
Age (years)	69 ± 9	70 ± 9
Gender (%F)	50%	49%
BMI (kg m^−2^)	30 ± 5	29 ± 5
Initial aHKA	–1.2° ± 2.4°	–0.9° ± 2.5°
Final aHKA	–2.0° ± 2.4°	–2.3° ± 2.2°
Initial extension	3.8° ± 3.7°	3.6° ± 4.6°
Initial flexion	132° ± 10°	127° ± 8°

Abbreviations: aHKA, arithmetic hip knee ankle angle; FJS, Forgotten Joint Score.

**Figure 8:**
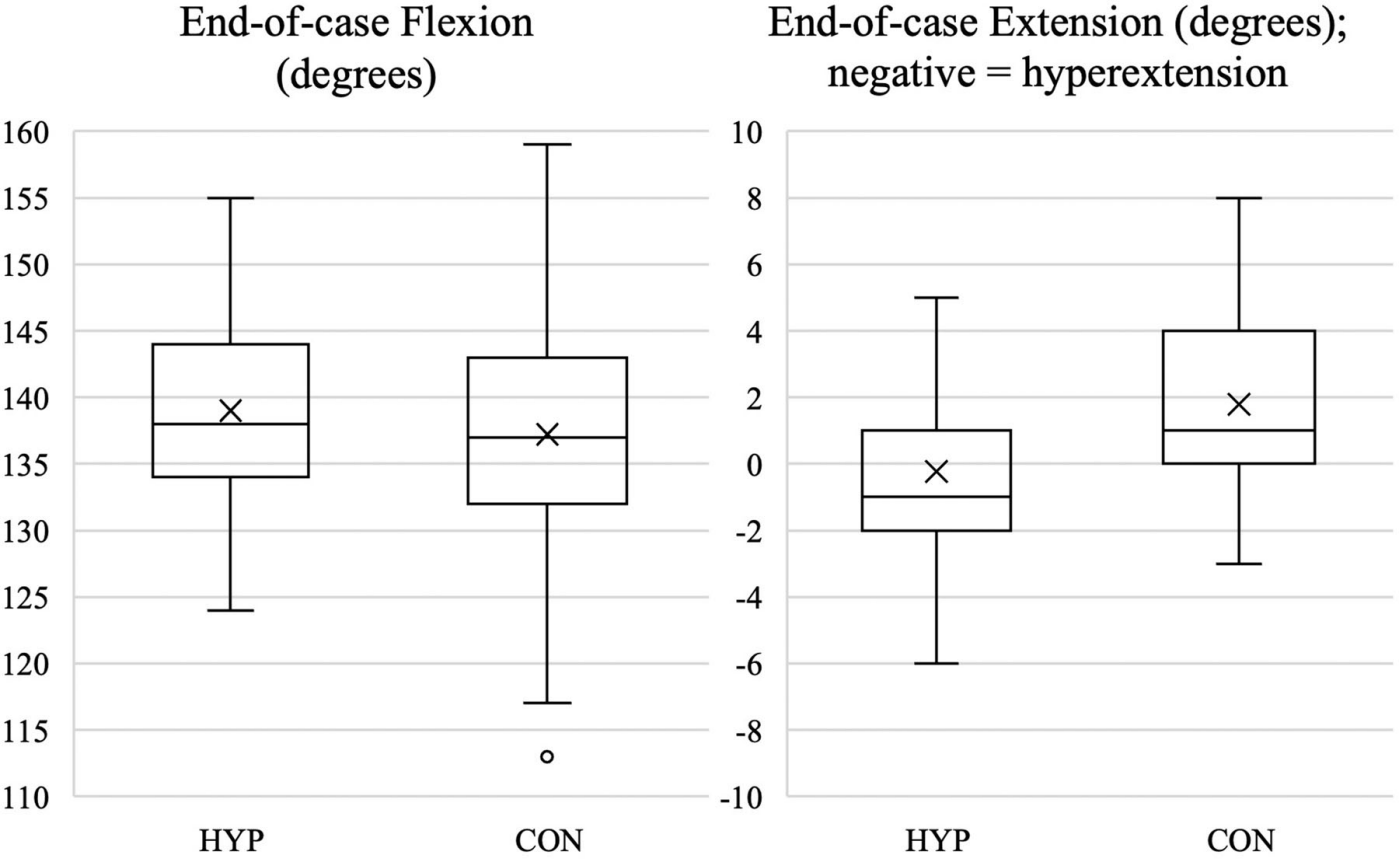
Box‐and‐whisker plot of the end‐of‐case robotically measured flexion and extension range. CON, control group; HYP, hyperextension group.

**Figure 9:**
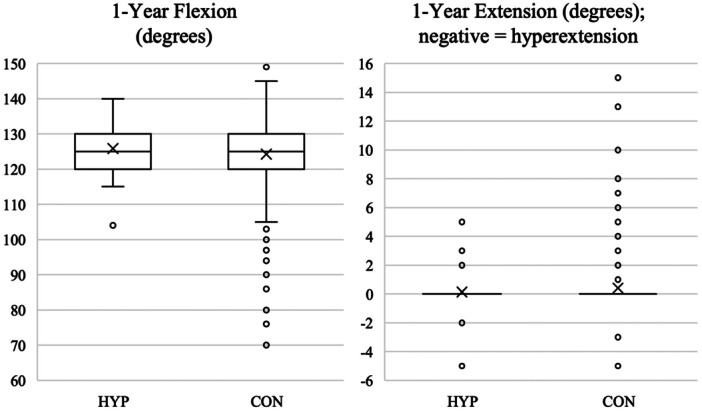
Box‐and‐whisker plot of the 1‐year goniometer flexion and extension range of motion. CON, control group; HYP, hyperextension group.

Although the hyperextension group had a greater end‐of‐case knee extension (mean –0.2° vs. 1.8°) and flexion (mean 139.0° vs 137.2°), it was commensurate with the noted cohort differences pre‐operatively (Figure 8). 1‐year range of motion data was available for 77% of cases. No significant differences were noted at 1‐year post‐operatively between groups although there was a trend towards less extension in the control group (flexion *p* = 0.12, extension *p* = 0.08, Figure 9).

For extension, hyperextension patients had a mean of 0.1° of fixed flexion (SD 1.1; 95% CI –2.2° to 2.5°) and control patients had a mean of 0.4° (SD 1.4; 95% CI –2.3° to 3.1°). In flexion hyperextension patients had a mean of 125.9° (SD 6.8; 95% CI 112.6°–139.2°) and control patients had a mean of 124.2° (SD 7.7; 95% CI 109.1°–139.5°). At one year, the maximum hyperextension recurrence recorded across groups was –5° (Figure 9).

## DISCUSSION

Patients presenting with hyperextension were a unique cohort in several ways, demonstrating 5° more pre‐operative knee flexion (Figure 1), less varus aHKA and a lower BMI (Table 1). Most patients in the hyperextension cohort had 5°–10° of hyperextension with only 10 patients having 10° or more (Figure 2). Only two of these patients recorded hyperextension at 1‐year review both of which were less than 5° (Figure 9) demonstrating that the extension balance was maintained. This was in keeping with previous studies demonstrating low rates of deformity recurrence [16, 17, 18].

The hyperextension patients follow the same trend of gap laxities as those in the control group, decreasing in size from lateral flexion, lateral extension, medial extension to medial flexion (Figure 3). The hyperextension cohort had gap values that were greater in size by up to 0.7–1.2 mm across all four measurements, demonstrating that hyperextension deformity is not an isolated extension gap problem. Through a functional alignment workflow both cohorts arrived at a similar gap outcome after balancing and implantation (Figure 4).

Contrary to traditional guidance, we observed that femoral joint‐line distalisation was small on average (+0.3 mm HYP vs. CON) and that 93% of HYP knees had <2 mm distalisation (Figures 5 and 6). These findings align with prior work by Seo et al. showing minimal change in distal femoral resection in hyperextension cohorts [19]. In this cohort surgeons chose to balance the through‐range laxity by elevating the tibial joint line as a combination of under‐resection of bone and increased liner thickness (Figure 5). The alternative to this would be distalising the femur, decreasing the tibial joint line elevation and then closing the flexion gap by increasing the size of, or posteriorly translating, the femoral component. This alters joint kinematics and there is emerging evidence to suggest that over‐sizing the femoral component in TKA may be detrimental to revision rates [2]. In keeping with our findings that the gap laxity is a through range phenomenon, we demonstrate that a functional alignment balancing technique results in most changes to decrease gap size occurring on the tibial side (Figure 5). There were no differences observed in PROMS or range of motion at the 1‐year mark (Figures 7, 8, 9).

The key strength of this study is the integration of 1‐year functional outcome data with high quality pre‐ and intra‐operative numerical data on range of motion and quantitative gap laxities, facilitating a novel analysis. The main limitation is that due to the infrequent nature of knee hyperextension the cohort sizes were disparate, and a greater number of hyperextension patients may have offered more opportunity for subgroup analyses. Although the intra‐operative start‐of‐case range of motion figures were CT‐based from the robotic system, the 1‐year range of motion data were captured using goniometer measurement which has accuracy limitations and human bias, however a practical and accessible alternative does not exist. The potential for clustering, missingness bias and using a 2 mm cartilage assumption are further limitations.

Generalisability is also somewhat limited due to the study analysing a single implant family and robotic workflow combination and future studies using other systems would provide valuable contribution to the literature. There was no comparator group where routine distalisation did occur and thus it is not possible to confirm if the method presented here has better outcomes than routine under‐resection of the distal femur. The authors believe that preservation of distal femoral anatomy is important for functional knee kinematics and to avoid mid‐flexion instability. Further research could examine whether the same phenomenon observed here applies to patients presenting with fixed flexion deformity and to undertake research looking at the different patterns of gap laxity and their association with outcomes.

## CONCLUSION

Hyperextending arthritic knees have increased laxity throughout the range of motion. Minimal joint line distalisation of an average of 0.3 mm may be sufficient to balance these knees and deliver good functional outcomes without hyper‐extension recurrence at 1 year. The use of functional alignment to assess soft tissue balance prior to bone resection demonstrates that these knees have a global laxity that may be successfully managed with predominantly tibial changes.

## AUTHOR CONTRIBUTIONS

**Anton Lambers**: Methodology; formal analysis; investigation; data curation; writing–original draft; writing–reviewing and editing. **Serene Lee**: Formal analysis; data curation; writing–reviewing and editing. **Kate Langton**: Formal analysis; data curation. **Dermot Collopy**: Writing–review and editing; conceptualisation; investigation; supervision; resources. **Gavin W. Clark**: Conceptualisation; writing–review and editing; investigation; methodology; supervision; resources; project administration.

## CONFLICT OF INTEREST STATEMENT

D. Collopy reports grants or contracts, consulting fees, and participation on a data safety monitoring board or advisory board from Stryker, as well as payment or honoraria for lectures, presentations, speakers bureaus, manuscript writing or educational events from Stryker, Zimmer Biomet, and AO Recon, all of which were unrelated to this study. G. W. Clark reports grants or contracts, consulting fees, payment or honoraria for lectures, presentations, speakers bureaus, manuscript writing or educational events, and support for attending meetings and/or travel from Stryker, unrelated to this study. A. Lambers has completed a robotic arthroplasty fellowship funded by Stryker. The remaining authors declare no conflicts of interest.

## ETHICS STATEMENT

Ethics approval was granted by the hospital group HREC (SJGHC HREC ref1388).

## Supporting information

Appendix A.

## Data Availability

Data are available on request from the authors.
