# Supplementary material for: Knee hyperextension is a sign of through‐range laxity and may be effectively managed without routine joint line distalisation in a robotic functional alignment workflow
Source: Knee Surg Sports Traumatol Arthrosc. 2025 Dec 1;34(8):2906–15. doi: 10.1002/ksa.70203 (PMC13418321; doi:10.1002/ksa.70203)
Supplement: Supplementary file 1 — Appendix A. [file KSA-34-2906-s001.docx]

**Appendix A – Severe (10 degrees or greater) Hyperextension Cases**

| **TABLE I**: Start-of-case values | | | | | | | | | | |
| --- | --- | --- | --- | --- | --- | --- | --- | --- | --- | --- |
| Case | BMI | Age | Gender | Extension | Flexion | Alignment | Ext Gap (L) | Ext Gap (M) | Flex Gap (L) | Flex Gap (M) |
| 1 | 35.0 | 61 | Female | –15 | 127 | –5.5 | 4.5 | 3.5 | 3.0 | 0.0 |
| 2 | 32.0 | 62 | Female | –13 | 143 | 0.0 | 7.0 | 6.0 | 7.0 | 4.0 |
| 3 | 35.0 | 60 | Female | –13 | 128 | –12.0 | 5.0 | 3.0 | 4.0 | 0.0 |
| 4 | 29.3 | 53 | Male | –12 | 130 | –3.0 | 3.0 | 4.0 | 5.0 | 2.0 |
| 5 | 24.1 | 78 | Male | –12 | 134 | –10.0 | 5.0 | 4.5 | 6.0 | 1.5 |
| 6 | 25.0 | 65 | Male | –11 | 151 | –11.0 | 5.0 | 6.0 | 8.0 | 4.0 |
| 7 | 31.0 | 86 | Female | –11 | 151 | –3.0 | 4.0 | 4.0 | 6.0 | 4.0 |
| 8 | 40.0 | 50 | Female | –11 | 134 | –3.0 | 6.0 | 6.0 | 9.0 | 1.0 |
| 9 | 27.0 | 62 | Male | –11 | 143 | 0.0 | 6.0 | 4.5 | 5.0 | 3.0 |
| 10 | 29.0 | 63 | Male | –10 | 137 | –11.0 | 3.5 | 3.0 | 7.5 | 2.0 |

| **TABLE II**: End-of-case values | | |  |  |  |  |  |
| --- | --- | --- | --- | --- | --- | --- | --- |
| Case | Extension | Flexion | Alignment | Ext Gap (L) | Ext Gap (M) | Ext Gap (L) | Flex Gap (M) |
| 1 | –2 | 131 | –3.5 | 3.0 | 1.0 | 1.0 | 0.5 |
| 2 | –2 | 143 | –2.0 | 5.0 | 5.0 | 5.0 | 4.0 |
| 3 | –1 | 135 | –6.0 | 7.0 | 5.0 | 6.0 | 5.0 |
| 4 | –4 | 141 | –1.0 | 7.0 | 6.0 | 7.0 | 6.0 |
| 5 | 3 | 133 | –6.0 | 1.0 | 0.5 | 3.0 | 0.5 |
| 6 | –1 | 147 | –2.0 | 5.0 | 5.0 | 9.0 | 5.0 |
| 7 | 0 | 152 | –1.0 | 4.0 | 4.0 | 5.0 | 4.0 |
| 8 | –4 | 131 | 0.0 | 5.0 | 5.0 | 6.0 | 4.0 |
| 9 | –2 | 147 | –2.0 | 2.0 | 2.0 | 3.0 | 2.0 |
| 10 | –6 | 140 | –5.0 | 3.5 | 2.5 | 7.0 | 2.5 |

| **TABLE III**: Changes in Joint Line | | |  |  |
| --- | --- | --- | --- | --- |
| Case | Change Femur Joint Line | Change Post Cond Offset | Change Tibial Height | Poly |
| 1 | 1.3 | –1.5 | 5.0 | 12 |
| 2 | 0.0 | –1.3 | 5.5 | 11 |
| 3 | 0.0 | –2.3 | 8.0 | 14 |
| 4 | 0.5 | –0.5 | 5.0 | 13 |
| 5 | 0.5 | –1.3 | 7.5 | 13 |
| 6 | 0.5 | –1.0 | 4.5 | 11 |
| 7 | 0.0 | 0.0 | 4.8 | 11 |
| 8 | 0.0 | –1.5 | 5.8 | 12 |
| 9 | 0.0 | –1.5 | 5.0 | 12 |
| 10 | 0.0 | –0.3 | 3.0 | 11 |

| **TABLE IV:** Mean Start-of-case Gaps by Group | | | |
| --- | --- | --- | --- |
|  | **>10' HYP** | **HYP** | **CON** |
| Flexion Lateral | 6.1 | 5.2 | 4.5 |
| Extension Lateral | 4.9 | 4.9 | 3.8 |
| Extension Medial | 4.5 | 4.2 | 3.0 |
| Flexion Medial | 2.2 | 1.8 | 1.1 |

| **TABLE V:** Mean End-of-case Gaps by Group | | | |
| --- | --- | --- | --- |
|  | **>10' HYP** | **HYP** | **CON** |
| Flexion Lateral | 4.0 | 3.2 | 3.5 |
| Extension Lateral | 2.5 | 2.3 | 2.2 |
| Extension Medial | 1.8 | 2.1 | 2.1 |
| Flexion Medial | 1.6 | 1.6 | 1.8 |
